# Patient Education During Hospitalization From the Perspective of Nurse Managers: A Qualitative Study

**DOI:** 10.1111/nhs.70052

**Published:** 2025-02-02

**Authors:** Jessica Longhini, Elisa Ambrosi, Barbara Tescaro, Noemi Derugna, Maria Luisa Ferro, Federica Canzan

**Affiliations:** ^1^ Department of Diagnostics and Public Health University of Verona Verona Italy; ^2^ Local Health Authority Berica Vicenza Italy

**Keywords:** hospital, multidisciplinary, nurse management, patient education, self‐care

## Abstract

The aim of this study was to explore patient education in surgical and medical wards from the perspective of nurse managers. A qualitative descriptive study was conducted with 28 nurse managers from 34 wards in two hospitals, using content analysis. Three themes were identified. The first “Characteristics of patient education” highlights the definitions, goals, and interdisciplinary nature of patient education, emphasizing its role in promoting autonomy and safe discharge. The second “Delivering patient education” focuses on the content, methods, and timing of education. While some managers supported routine care integration, others preferred dedicated sessions, highlighting a need for flexibility in approaches. The third “Evaluating and Improving Patient Education” examines assessment strategies, outcome tracking, and quality maintenance. Effective patient education was linked to reduced readmissions and fewer support calls. Documentation was seen as both essential and burdensome. The study underscores the complexity and benefits of patient education while addressing challenges like time constraints and workload. Recommended strategies include staff training, developing educational tools, structured but flexible approaches, enhanced documentation, and fostering interprofessional collaboration.

**Summary**

- Despite available literature and recommendations, time constraints and high workloads still limit patient education, focusing mainly on technical information rather than embedding psychosocial support.
- Effective patient education requires structured approaches mixed with personalized approaches and training in both clinical and educational skills, including the use of digital tools, multimedia resources, and strategies targeting older people.
- There is an urgent need for various stakeholders to include patient education within the hospital priorities, enhancing training and documentation, and fostering collaboration between hospitals and primary care to reduce readmissions.

## 1 Introduction

Self‐care abilities are fundamental when people are affected by acute and chronic conditions as they need to cope with transforming changes in their body, identity, and daily life (Riegel, Jaarsma, and Strömberg 2012; World Health Organization 2021). Patient education, especially therapeutic patient education, aims to improve clinical outcomes and quality of life by empowering patients to manage their conditions through self‐care. Indeed, patient education is defined as “a process of assisting consumers of health care to learn how to incorporate health‐related behaviours (knowledge, skill, attitude) into everyday life with the purpose of achieving the goal of optimal health” (Bastable 2017). According to the World Health Organization, it involves educational interventions by different trained health professionals or expert patients to help people and their families understand and accept their conditions, set goals, and acquire the necessary knowledge, attitudes, and skills. The goal is to help patients balance their lives with their illness, prevent complications, and achieve optimal outcomes, shifting their perspective from illness to wellness (World Health Organization 2023). Recent systematic reviews have concluded that patient education effectively improves physical activity levels, eating habits, disease knowledge, functional skills, quality of life, and health outcomes such as blood pressure, glycosylated hemoglobin, and rehospitalizations (Kim, Park, and Song 2021; Lee et al. 2022; Pigott et al. 2022; Zhao et al. 2021).

Patient education is crucial in hospitals for teaching self‐care. However, patients have reported being poorly informed, especially at the discharge, and complained of not being educated on how to self‐manage their condition and when searching for appropriate care for red‐flag signs of decompensation (Marca‐Frances et al. 2020; Schjodt et al. 2022; Trivedi et al. 2023). Missing education also regards medication plans, sleep disturbances, sex life, symptoms, changes in appetite and urination, pain and fatigue management, and when to resume work (Blöndal, Sveinsdóttir, and Ingadottir 2022; Trivedi et al. 2023). Poor patient education hampers high‐quality care, leading to misunderstandings of postdischarge care, poor medication adherence, inadequate self‐care, and higher rates of adverse events and readmissions, especially among geriatric patients with polypharmacy (Marca‐Frances et al. 2020; Mortelmans et al. 2021; Schjodt et al. 2022; Trivedi et al. 2023).

As a consequence, previous studies have reported a need to improve patient education during hospitalization (Krook, Iwarzon, and Siouta 2020; Trivedi et al. 2023). Hospitals pose challenges due to acute conditions, brief patient interactions, and time constraints (Boyde, Tuckett, and Ty 2021; Pueyo‐Garrigues et al. 2022; See et al. 2020; Wang et al. 2024). Healthcare professionals find difficulties in providing patient education due to the excessive workload, rendering it one of the most neglected forms of care (Boyde, Tuckett, and Ty 2021; Pueyo‐Garrigues et al. 2022; See et al. 2020; Wang et al. 2024).

### 1.1 Nurse Managers' Role in Patient Education

Given the multidisciplinary nature and organizational complexities of patient education, nurse managers hold a distinctive position that enhances understanding of this critical aspect in hospital wards. Positioned at the intersection of clinical practice, policy implementation, and interprofessional collaboration, nurse managers play a pivotal role in coordinating cohesive care strategies among healthcare professionals (González‐García et al. 2021). This strategic positioning enables them to evaluate the contributions of various professionals to patient education and identify communication or procedural gaps that may hinder patient understanding and adherence to treatment plans (González‐García et al. 2021; McNamara et al. 2011).

Nurse managers are also acutely aware of practical challenges, such as staffing shortages and time constraints, which directly impact the quality of patient education (Nurmeksela et al. 2021; See et al. 2020). Their perspectives are instrumental in setting educational priorities, allocating resources effectively, and aligning staff across specialties to deliver consistent and high‐quality patient education (Ghorbani et al. 2019; Kwame and Petrucka 2021; Wang et al. 2024). By leveraging their unique perspective, nurse managers can develop sustainable strategies to bridge interprofessional gaps, enhance resource allocation, and help integrate patient education practices into routine care. Such efforts align with the overarching goal of empowering patients to manage their health and engage in self‐care (Bastable 2017; World Health Organization 2023).

Despite their critical role, the perspectives of nurse managers on patient education have received limited attention in the literature. To date, we identified two papers only elucidating the perspective of nurse managers. Ghorbani et al. (2019) conducted a survey with 91 nurse managers to investigate mechanisms for involving nurses in patient education. See et al. interviewed 21 nurses at varying levels of practice, including only four nurse managers, focusing on patient education in postoperative care, primarily emphasizing the role of ward nurses as educators.

These studies reveal significant limitations. Both primarily examine nursing perspectives, neglecting the inherently multidisciplinary nature of patient education (Ghorbani et al. 2019; See et al. 2020). Additionally, their scope is narrow, addressing specific areas such as strategies to engage nurses in education (Ghorbani et al. 2019) or postoperative care (See et al. 2020). Furthermore, the qualitative component of See et al.'s research included only a small subset of nurse managers, limiting the depth of insights into their strategic role in patient education.

Unlike clinical staff, whose primary focus is direct patient care, nurse managers offer a broader, systems‐oriented perspective that encompasses resource allocation, policy adherence, and fostering interprofessional collaboration. However, research exploring this unique viewpoint remains scarce.

### 1.2 Study Objective

This study aims to fill this gap by examining patient education practices for adult patients hospitalized in surgical and medical wards from the perspective of nurse managers.

## 2 Methods

### 2.1 Research Questions

Scrutiny of the available literature on the topic helped us to identify the following research questions:

- How do nurse managers perceive the current quality and implementation of patient education in hospital wards, including the barriers and facilitators to its effective delivery?
- What strategies do nurse managers employ or recommend to enhance therapeutic patient education in hospital wards?

### 2.2 Design and Setting

We conducted a qualitative descriptive study, guided by the methodologies outlined by Kim, Sefcik, and Bradway (2017), Sandelowski (2000), in two hospitals in Northern Italy between December 2023 and April 2024. The study is reported according to the Standards for Reporting Qualitative Research (SRQR) (O'Brien et al. 2014). The two hospitals were selected to ensure representation of institutions of varying sizes and geographic contexts: One hospital located in a larger city and the other in a smaller city with a rural catchment area. This selection aimed to capture a diverse range of experiences and practices, thereby providing a more comprehensive understanding of the research topic. In these hospitals, patients are admitted to the wards through multiple pathways, including self‐referral from home, emergency department visits, transfers from nursing homes, referrals by general practitioners, or elective surgery prescribed by specialist physicians. Notably, no other health or social professionals are authorized to prescribe hospital admission or emergency department visits.

At the ward level, nurse managers in these hospitals have responsibilities encompassing various domains, including planning and management of professional/work activities, coordination and execution of innovative projects, personnel management, staff development and training, fostering collaborative and interprofessional relationships, management of material resources and technologies, and oversight safety measures. This scope of responsibilities highlights the multifaceted role of nurse managers in ensuring effective ward‐level operations and contributing to patient care and education.

### 2.3 Sample

We recruited nurse managers through a maximum variation convenience sampling. Inclusion criteria required participants to be nurse managers in a medical or surgical ward. Managers of pediatric, intensive care, and psychiatric wards were excluded due to the unique characteristics and patient education frameworks specific to these contexts, which differ from those in medical and surgical wards.

Nurse managers were selected for their pivotal role in coordinating ward staff, including nurses, nursing aides, and healthcare professionals such as physiotherapists, who often work across multiple wards. This central role provides nurse managers with a unique vantage point to observe, assess, and support care processes provided by diverse health professionals. Furthermore, their involvement in organizational activities positions them as privileged observers of patient education practices, enabling them to identify factors that facilitate or hinder effective education delivery and to develop strategies to enhance these processes within hospital wards.

A member of the research team initiated the recruitment process by contacting the hospital manager to identify eligible nurse managers overseeing medical and surgical wards. These nurse managers were subsequently invited to participate in the study through institutional emails containing detailed information about the research objectives and procedures. Interested participants scheduled interviews at their convenience. Informed consent was obtained from each participant at the beginning of the interview. Participant recruitment continued until data saturation was reached in line with the guidance of Saunders et al. (2018). Data saturation was achieved when no new insights emerged, and analysis of the interviews revealed redundancy in the results. To ensure saturation had been reached, two additional interviews were conducted, confirming that no further data collection was required.

### 2.4 Data Collection

Semistructured face‐to‐face interviews were conducted in a dedicated room in the ward of the manager after working hours and where they could not be disturbed or listened to by the staff. Interviews were conducted by two trained nurses with a master's degree in family and community nursing and a PhD research fellow following a semistructured guide. All interviewers were unknown to the participants. The guide has been built based on the literature on the topic (Blöndal, Sveinsdóttir, and Ingadottir 2022; Ghorbani et al. 2019; Pueyo‐Garrigues et al. 2022; Trivedi et al. 2023; Wang et al. 2024) and the experience of the researchers. The interview guide is provided in Appendix A. Before starting with questions, we collected data related to the age, gender, and professional characteristics of participants.

### 2.5 Data Analysis

Interviews lasted from 31 to 94 min and were audio‐recorded and transcribed verbatim. A content analysis (Elo and Kyngäs 2008) was performed involving three researchers to ensure reliability and trustworthiness (Lincoln and Guba 1985). Researchers conducted multiple readings of the transcriptions to familiarize them with the source material. Then, units of meaning were identified according to the aim of the study to obtain codes, avoiding missing relevant information, by two researchers independently for each interview (Lindgren, Lundman, and Graneheim 2020). Then, the independent analysis was compared, and a consensus was reached in grouping codes with similar meanings in subthemes that, in turn, were clustered in themes.

### 2.6 Rigor and Trustworthiness

To ensure rigor and trustworthiness, several strategies were employed, including maintaining researcher neutrality, practicing reflexivity, and validating findings through feedback from academic and clinical colleagues.

#### 2.6.1 Maintaining Researcher Neutrality

The researchers involved in data collection and analysis had no prior relationship with the participants. This ensured an objective approach by minimizing the risk of interpreting participants' answers based on preconceived notions or personal knowledge.

#### 2.6.2 Practicing Reflexivity

Reflexivity, a critical element in qualitative research, was integral to this study. It was employed to identify and mitigate potential biases stemming from the researchers' prior clinical experiences with patient education. Reflexivity is essential in qualitative research as it allows researchers to systematically examine their positionality, assumptions, and potential influence on the research process and outcomes (Olmos‐Vega et al. 2022). Two key methods were used to embed reflexivity in the research process. First, each researcher maintained a reflexive journal, documenting their assumptions, emotional reactions, and reflections during data collection and analysis. This journaling facilitated the identification of potential biases and unconsciously influence on their interpretations, thereby promoting a more deliberate and balanced analysis. Reflexive journaling is recognized as a powerful tool to bring subconscious biases to light, supporting greater analytical rigor (Olmos‐Vega et al. 2022). Additionally, regular collaborative meetings among the research team further reinforced reflexivity. During these meetings, team members critically examined each other's reflections and interpretations, challenging assumptions and encouraging alternative perspectives. These discussions fostered a collective reflexive stance, ensuring accountability to both the data and the research team.

#### 2.6.3 Validating Findings

To enhance the validity of the findings, member checking and peer feedback were conducted. Academic and clinical colleagues familiar with the study context, but not directly involved in data collection, reviewed the identified themes and subthemes. Their feedback helped confirm the plausibility, coherence, and relevance of the interpretations, ensuring the findings accurately reflected the participants' experiences.

### 2.7 Ethical Aspect

The study has been approved by the Comitato di Approvazione della Ricerca sulla Persona (N. 28_2023). Signed informed consent was obtained from participants by researchers prior to conducting the interview. Data have been managed anonymously, ensuring privacy criteria according to GDPR.

## 3 Results

### 3.1 Characteristics of Participants

Twenty‐eight nurse managers coordinating 34 wards and 874 health professionals were interviewed. The majority were women (24, 86%) with an average age of 46 years (IQR, 39; 55 years). At the time of the interviews, 53.6% of participants had over 10 years of experience as nurse managers, while 26% had less than 5 years (Table 1).

**TABLE 1:** Characteristics of participants.

	Total = 28
	Median (IQR)
Age, years (IQR)	46 (39; 55)
Women	24 (86)
Experience as a nurse (years)	12 (10; 15), min = 3; max = 30
Experience as a nurse manager (years)	10 (4, 75; 17), min = 0,5; max = 25
Number of professionals managed	31 (39; 21), min = 20; max = 55
Nurses	23 (min = 15, max = 33)
Nurses' aides	11 (min = 5, max = 18)
Physiotherapists^a^	2 (min = 1, max = 4)
Occupational therapist^a^	2 (min = 1, max = 3)
Dieticians^a^	2 (min = 1, max = 3)

Wards managed, *N*	Tot = 34	Bed‐size	*N*. nurse managers^b^
	*N* (%)	Median	Median
Surgical wards	16 (47)	20 (min = 11, max = 41)	12 (of 28, 43%)
Orthopedics and rehabilitation	2		
Ophthalmology	1		
Neurosurgery	2		
Cardiac surgery	2		
Gynecology	2		
General surgery	3		
Vascular surgery	1		
Maxillofacial surgery	1		
Plastic surgery	2		
Medical wards	18 (53)	25 (min = 13, max = 53)	16 (of 28, 57%)
Hematology	2		
Oncology	2		
Geriatrics	3		
Neurology	2		
Infectious diseases	1		
Nephrology	1		
Pneumology	2		
Urology	1		
Cardiology	2		
Internal medicine	3		

^a^ These health professionals are shared across wards of the same department.

^b^ Some nurse managers coordinated more wards.

### 3.2 Nurse Managers' Perspective on Patient Education

The analysis of the interviews revealed three overarching themes: (1) Characteristics of patient education, (2) Delivering patient education, and (3) Evaluating and improving patient education. Each theme is further organized into subthemes (Table 2).

**TABLE 2:** Subthemes and themes.

Themes	Subthemes
Characteristics of patient education	Definition of patient education
	Aims and benefits of patient education
Delivering patient education	Contents and methodology
	Timing
	Tracking and reporting
Evaluating and improving patient education	Judging the quality and measuring the impact
	Hindering and facilitating factors
	Strategies to promote patient education

#### 3.2.1 Theme 1. Characteristics of Patient Education

The first theme encompasses two subthemes, called “Definition of patient education” and “Aims and benefits of patient education.” Participants described patient education as a continuous, collaborative process designed to foster autonomy, skill acquisition, and stability during hospitalization and in the home setting. They highlighted that this process is primarily delivered by nurses and physiotherapists. Participants consistently emphasized the importance of establishing a therapeutic alliance and tailoring interventions to individual needs. They noted that personalized patient education contributes to improve health outcomes, a reduction complication, and lower healthcare costs.

##### 3.2.1.1 Definition of Patient Education

Seven nurse managers (25%, six from medical wards) provided a specific definition of patient education, while the remaining 21 reported different components to describe the concept. It was described as a process of patient or family education that, through the collection of information (family background, disease awareness, etc.), promotes the learning of skills and the maintenance of psychophysical stability during hospitalization and upon returning home. It is part of professionals' activities and is a continuous process, starting preferably upon admission to the ward and continuing until achieving the highest possible autonomy.

Education is a process that allows us to investigate knowledge and awareness and allows the patient to maintain their autonomy and cognitive state and to acquire new skills, to guarantee a safe return home and avoid re‐hospitalization, with consequent decrease in healthcare costs. (ID4, Medical ward).

In addition, patient education was defined as a process in which the patient must be involved and aware that the effectiveness of education is also his/her responsibility, as well as of the professional.

According to all interviews, the most involved figures in education were the nurse and physiotherapist, even though all professionals, including physicians, took part in patient education. The recipients were the patient, their children, spouse, other family members, private informal caregivers, and friends. All participants agreed that the informal caregiver may not necessarily be a family member. A manager from a medical ward defined “family” as whoever directly assists the patient, regardless of blood ties.

##### 3.2.1.2 Aims and Benefits of Patient Education

According to 25 managers (89%, 14 from medical wards), the objectives of patient education delivered during hospitalizations referred to the promotion of autonomy and safety at home. Undergoing surgery or facing hospitalization may lead to a change in the daily routine, which can be challenging for the patient and family; early education allows them to process the received information, become aware of the condition, acquire technical skills, and consequently return home with greater confidence and less worry.

Changing habits after surgery can be difficult for the patient; early education allows him to process the information received and prepare for what he will have to face after the operation. (ID11, Surgical ward).

Managing the condition at home is different than in the hospital. Education allows the patient and caregiver to acquire autonomy at home and be discharged safely. (ID19, Medical ward).

Patient education aimed to establish a therapeutic alliance with patients and families, supporting them while maintaining and enhancing their autonomy, skills, and self‐care. The goal was to enable them to independently manage their needs, improve their lifestyle, and maintain a good quality of life despite illness. Personalized educational interventions addressed these needs, aiming for long‐term objectives such as preventing disease recurrence and recognizing when additional support is necessary. Eighteen managers (64%, 11 from medical wards) observed that this approach reduces calls to hospital wards for information, emergency service use, and hospital readmissions, lowering healthcare costs, alleviating stress, and preventing complications like spreading resistant microorganisms among patients.

#### 3.2.2 Theme 2. Delivering Patient Education

The second theme includes three subthemes, namely, “Contents and methodology,” “Timing,” and “Tracking and reporting.” Participants consistently emphasized team‐based and patient‐centered approaches. However, perspectives diverged on the implementation of education, with some managers viewing it as an integrated and routine aspect of care, while others advocated for structured and dedicated sessions. Similarly, opinions on documentation were mixed, with some managers seeing it as essential for quality and accountability, while others perceived it as an administrative burden.

##### 3.2.2.1 Contents and Methodology

Education content in hospital wards varied based on specificities like patient characteristics and whether they are in medical or surgical areas. All nurse managers from surgical departments focused on managing devices (such as urinary catheters, stomas, and drains), surgical wound care, and pre‐ and postoperative behaviors to prevent and manage complications after discharge. According to nurse managers from medical wards, patient education covers a wide range of topics, including mobilization, pressure ulcer prevention and management, nutrition, dysphagia management, promotion of regular bowel movements, absorption aids, and home oxygen therapy. Patients across all wards received education on oral and insulin therapy management. Educational interventions were typically team‐based and tailored to patient needs, according to all managers. Methods included the teach‐back or verbal explanation, “show me” techniques for technical skills, directly observed therapy (DOT), and reading discharge letters with patients. Before COVID‐19, three managers (11%, two from medical wards) reported having used peer tutoring meetings, meant as structured moments where expert patients from the community meet patients with a new diagnosis or poor education on the disease. Two managers from surgical wards narrated that patient with longer hospital stay for rehabilitation (3 months–1 year) could obtain therapeutic permits, where they can apply learned skills at home. Twenty‐three managers (82%, 13 from medical wards) described the use of tools like personalized therapeutic schemes, charts, and informative brochures (from wards or companies) to support education, encouraging patients to ask the staff questions and provide ongoing informational support at home.

The brochure allows staff to start the educational intervention early and the patient to acquire medical language to facilitate the understanding of the information given to him by the staff. Furthermore, it is a useful support tool that remains for him when the patient is at home. (ID17, Surgical ward).

##### 3.2.2.2 Timing

Managers unanimously agreed on the importance of early and continuous patient education throughout all phases and settings of hospitalization. However, the timing of education—specifically, when and how long it occurs—was influenced by several factors. These included the reason for hospitalization, duration of stay, patient and caregiver characteristics, their level of commitment to education, specific educational needs, and organizational requirements.

For scheduled admissions, all managers from surgical wards reported that patient education, meant as transmission of information, begins during the preadmission visits. Ten of 18 managers of medical wards reported that patients with specific needs, such as older patients or those requiring intensive educational interventions, received ongoing education throughout their hospitalization. All managers from medical and surgical wards highlighted those technical skills were typically taught near discharge, with some patients receiving preoperative education.

Beyond these cases, all managers reported that professionals utilize any phase of the work shift for patient education, tailored to the type of intervention needed. Caregiver education mainly occurs during visiting hours or by appointment. Educational sessions can range from 10 min to 2 h and may be repeated over several days.

Conflicting opinions emerged regarding how educational activities are integrated into the patient management plan; 21 managers (75%, 13 from medical wards) described any interaction with the patient as an educational moment and, therefore, asserted that this practice is part of routine activities and is carried out daily.

Education is continuous in every phase and setting of hospitalization and is integrated into routine activities rather than performed in structured moments. (ID7, Medical ward).

The remaining seven managers (25%, four from surgical wards) agreed that education is a structured and dedicated event alongside routine activities and follows specific protocols. However, they highlighted the need for personalized plans by scheduling educational interventions at optimal times based on the patient's emotional state and clinical condition, and organizational aspects.

##### 3.2.2.3 Tracking and Reporting

Regarding tracking and reporting patient education, two main ways of thinking have emerged.

According to 20 managers (71%, 12 from medical wards), education was not discussed as a routine practice; therefore, documenting education activities would have purely legal significance and would not promote its implementation. In these contexts, protocols and checklists were not used because it is believed that education should be personalized and not standardized; moreover, bureaucracy would detract from time spent with the patient.

Requiring professionals to report in a detailed manner patient education would turn into a workload that could impact professionals' performance. Patient education is part of patient care; therefore, it's perceived as something obvious. (ID24, Medical ward).

Five managers (18%) from medical and surgical wards adopted a structured approach to track and report patient education. This included organizing dedicated meetings within multidisciplinary teams, using checklists and protocols, and incorporating specific sections in medical documentation to outline educational objectives, methods, and target recipients. In addition, three managers from medical wards reported that educational activities were logged in daily diaries, discharge information, or Situation, Background, Assessment, Recommendation (SBAR) reports, as there was not a designated section for education in medical records. However, these records often focused solely on the educator's perspective without detailing patient information, methods used, or time spent.

#### 3.2.3 Theme 3. Evaluating and Improving Patient Education

The third theme includes three subthemes, namely, “Judging the quality and measuring the impact,” “Hindering and facilitating factors,” and “Strategies to promote patient education.” Most managers rated patient education as good or high in quality. However, some noted a perceived lack of quality due to time constraints and the prioritization of clinical tasks. Commonly cited barriers included skill deficits, resource limitations, and organizational complexities, while facilitators involved motivated teams, effective documentation tools, and structured transitional care. Divergent views emerged on training approaches, with preferences divided between experiential and formal methods. Proposed strategies for enhancing patient education included increasing staffing levels, integrating education into routine care, leveraging multimedia tools, and fostering innovation by valuing ideas from new staff members.

##### 3.2.3.1 Judging the Quality and Measuring the Impact

Overall, nurse managers assessed the quality of patient education delivered by staff at a level ranging from sufficient to high. Seven managers (25%, five from medical wards) considered patient education insufficient due to the limited time available for education. Often, organizational needs, particularly workload and bed demand, took priority over educational activities, which were therefore delivered at discharge, consequently compromising its effectiveness. Six managers (21%, five from medical wards) stated that identifying patients' clinical needs is easier than identifying educational needs, leading to delays in starting educational interventions.

However, 21 managers (75%) equally distributed across medical and surgical wards reported a good or high quality of patient education even though they found it difficult to evaluate. They reported various factors influencing their judgment: the willingness to educate, the value attributed to education, the stability and level of training of the team, and the technical, communicative, and relational skills of the staff, which might vary among professionals and roles.

Nine managers (32%, six from medical wards) provided indicators of the quality of education, such as a reduced number of phone calls or patient visits for information, as well as fewer emergency department visits.

The high number of phone calls requesting information and the visits to the emergency room linked to difficulties in managing the device or medications are indicators of the poor quality of the education provided. (ID2, Medical ward).

##### 3.2.3.2 Hindering and Facilitating Factors

All managers agreed that patient education is threatened by challenges from individual, staff, and organizational factors. Personal traits, emotional states, and clinical complexities can impede smooth educational interactions. Aggressive behaviors toward staff, coupled with inadequate support networks and socioeconomic disparities, hinder educational opportunities. Additionally, reliance by patients on digital platforms for information dissemination can undermine trust and engagement with professionals. Conversely, formal support networks and motivated patients and caregivers foster therapeutic alliances. Within hospitals, institutional frameworks present challenges such as staff shortages and high workloads, limiting time for patient education. In addition, structuring education outside routine activities can stress staff and hinder integration with clinical practice. Early discharges and bureaucratic hurdles reduce patient involvement and educational opportunities, while reduced workloads and predictable hospitalizations facilitate educational interventions. The strategic use of documentation tools and informative materials could streamline educational planning.

For 17 managers (61%, 12 from medical wards), transitioning from hospital to home presented challenges that required meticulous planning amid limited resources and structured transitional care programs. Strengthening collaboration between healthcare and social services is crucial for addressing health needs and ensuring the continuity and effectiveness of educational efforts.

Personnel composition and disposition significantly influenced patient education efficacy according to 22 managers (78%, 12 from medical wards). High turnover rates and inadequate skill proficiency delayed identifying educational needs and limited the use of advanced methods like counseling. Deficiencies in technical, communicative, and relational competencies could also lead to staff reluctance in delivering educational activities.

The characteristics of the patient and/or caregiver influence the learning ability and the time taken to carry out education. Furthermore, the perception of a lack of technical, communication, and relational skills can lead professionals to not carry out education. (ID12, Medical ward).

Seven managers (25%, five from medical wards) also reported that the detachment of physicians from patient relationships exacerbates informational asymmetry, thwarting effective information exchange. In addition, they reported that a group of health professionals believing in the value of patient and family education and its link to patient well‐being is a vital source for educational activities.

The quality of education is influenced by the value that the staff attributes to it. (ID6, Medical ward).

Almost all managers (26, 93%, 15 from medical wards) sustained the need for managers to reinforce staff satisfaction with positive feedback on successful patient education outcomes to strengthen commitment to educational interventions. Regarding educational skill development, there was a divergence in training paradigms: 10 managers (36%, six from surgical wards) advocated for experiential learning, while the remaining preferred structured training by expert personnel.

##### 3.2.3.3 Strategies to Promote Patient Education

All managers suggested several strategies to enhance staff motivation and facilitate routine educational activities. These included increasing staff numbers, reducing workloads, integrating educational tasks into work plans, establishing dedicated educational spaces, and offering greater financial recognition. Twelve managers from medical wards (75%) and six from surgical wards (50%) emphasized the importance of having a mix of novice and experienced personnel for patient education during each shift.

Team‐based approaches were also recommended by 19 managers (68%, 12 from medical wards), reporting that collaborative discussions within teams help in the early planning of educational programs. Increasing involvement of non‐healthcare personnel, such as trained volunteers, was proposed to support activities like peer tutoring by 10 managers (36%, eight from medical wards).

Furthermore, 13 managers (46%, eight from medical wards) reported that introducing a case manager in each ward or several wards may be beneficial for organizing discharge pathways effectively. When a dedicated case manager is not available, training selected professionals to become mentors in patient education is advised.

Continuous education for healthcare professionals on patient education was reported as crucial for raising awareness and enhancing motivation according to all nurse managers.

Fifteen managers (54%, 10 from medical wards) advocated for creating dedicated spaces in healthcare documentation and using checklists and protocols to standardize education, although the remaining believed education should prioritize patient relationships and patients' needs over bureaucratic tasks.

Twenty‐four participants (86%), evenly distributed between medical and surgical wards, emphasized the importance of using various tools to support patient education. These tools include informational videos, tutorials, multilingual brochures, and posters displayed in the wards. They highlighted how patients generally prefer videos over brochures and stressed the need to expand the availability of videos in multiple languages. Digital platforms and mobile applications are also mentioned for disseminating educational content.

Eight managers (28.6%, five from medical wards) reported that nurse managers, when competent in patient education, should share their skills, train new staff, participate in educational planning, and provide feedback to boost team motivation. Twenty managers (71%, 12 from medical wards) emphasized the importance of valuing the innovative ideas of newly hired staff, particularly younger professionals, and those with expertise in digital tools.

Different strategies could be adopted to improve patient education in the future, including having time dedicated to education integrated into routine activities, making education traceable within clinical documentation, specific training courses on education, and motivating staff to carry out education. (ID22, Medical ward).

At the organizational level, focusing on families as the unit of care rather than solely as informal caregivers and increasing their participation in clinical pathways and ward activities could enhance education and address cultural or linguistic barriers according to 23 managers (82%, 13 from medical wards). Almost all participants (24, 85%) stressed the importance of identifying vulnerable patients early to involve informal caregivers or other professionals in delivering educational interventions. They advocated for using risk assessment tools and postdischarge care at home to reduce readmissions, reinforcing collaboration between hospitals and primary care through dedicated roles ensuring educational continuity.

## 4 Discussion

This study appears to be the first to comprehensively explore patient education across surgical and medical hospital wards from the perspective of nurse managers.

Patient education is seen as inclusive of families and informal caregivers, aiming to enhance outcomes such as reduced stress levels, lower rehospitalization rates, improved self‐care, and increased autonomy. These findings support existing research on the effectiveness of patient education (Marca‐Frances et al. 2020; Mortelmans et al. 2021; Schjodt et al. 2022; Trivedi et al. 2023).

A significant finding from our study is the limited attention given to the psychoemotional dimensions in patient education, such as self‐efficacy, motivation, and self‐identity. Instead, the focus predominantly lies on technical and informational aspects, often delivered through brochures or brief instructions. As such, the term “patient teaching” may be more appropriate than “patient education.” This aligns with findings from a previous study involving newly registered nurses, who reported that their patient education efforts were largely confined to providing information or instructions, with minimal integration of pedagogical techniques such as assessing learning needs, setting goals, and evaluating learning outcomes (Fawkes and Moore 2019). Our study highlights the critical importance of addressing psychoemotional aspects, mirroring the conclusions of Loyal et al. (2023) who emphasize the need for patient education to go beyond information transfer and focus psychological and behavioral readiness for health behavior changes.

In addition, our study showed that instructions are mostly limited to drug treatment, device management, or procedures, according to Spann et al. (2024) and, to a lesser extent, to lifestyles and decision‐making abilities, which are often crucial to avoid rehospitalizations in chronic disease as supported by Marca‐Frances et al. (2020). As stated by Riegel et al. (2022), promoting contextualized decision skills and transforming knowledge and skills into behaviors is fundamental to making patients and informal caregivers adequately self‐care in chronic conditions.

Our findings reveal that patient education is predominantly delivered opportunistically by health professionals during convenient moments, as reported by most managers. Conversely, a smaller proportion indicated that patient education is provided as a structured activity, particularly during elective admissions and for informal caregivers. This aligns with previous studies, which similarly observed that ward nurses often perform patient education in an unplanned manner, primarily during informal and spontaneous interactions with patients. These interactions frequently occur while assisting patients with daily living activities or administering medications (Fawkes and Moore 2019; See et al. 2020; Spann et al. 2024). These findings contribute to the ongoing debate about whether patient education should be formalized.

Several managers in our study highlighted the potential benefit of structured protocols and approaches, emphasizing that these could enable professionals to dedicate adequate and meaningful time to patient education. According to previous studies, the absence of formalized patient education frameworks often leads to its devaluation by nurses, reducing it to casual conversations with patients (Fawkes and Moore 2019; See et al. 2020; Spann et al. 2024). Additionally, participants in our study shed new light on the relevance of initiating patient education as early as possible in the care process, rather than reserving it primarily for discharge.

Managers noted that discharge‐based education is often ineffective, as it can result in increased readmissions and postdischarge calls seeking clarification or addressing missed information. These insights reinforce the value of incorporating structured patient education throughout the care continuum, starting at the earliest stages of treatment.

Previous studies showed that especially older adults perceived communication at the discharge as a one‐way conversation (Krook, Iwarzon, and Siouta 2020) and would be better informed and involved in decision‐making during the discharge process, elements that are sometimes missed (Marca‐Frances et al. 2020; Schjodt et al. 2022). A study interviewing hospitalized patients reported they struggled to understand diagnosis, symptoms, and treatment instructions and remember providers caring for them (Harrison et al. 2019; Marca‐Frances et al. 2020). This further complicates their ability to retain information or ask for clarifications (Harrison et al. 2019).

However, on the other side, participants of our study drew attention to the limits of the structured approach to patient education. Indeed, patient‐related and organizational factors can contribute to the avoidance or postponement of patient education.

Regarding patient characteristics, unstable clinical conditions and cognitive abilities can make patient education challenging. Previous studies have accordingly highlighted that illness severity, emotional state, motivation levels, and readiness to engage with healthcare professionals pose significant barriers to effective learning and active participation in care planning (Boyde, Tuckett, and Ty 2021; Fawkes and Moore 2019; Loyal et al. 2023). Our study highlights the importance of prioritizing patients' emotional and clinical conditions over predefined schedules to create an environment conducive to learning and, consequently, to enhance the value of informal patient education. Moreover, our managers identified several factors that may impede effective patient education, including patient's personal traits, aggressive behavior toward staff, lack of support networks, and socioeconomic disparities. These findings align with observations from previous studies (Fawkes and Moore 2019; Loyal et al. 2023). Regarding staff and organizational constraints, time limitations, heavy workloads, staff turnover, and short hospital stays often limit patient education and the application of advanced communication skills like structured counseling or motivational interviewing, in line with conclusions from earlier studies (Fawkes and Moore 2019; See et al. 2020; Temedda et al. 2024). Previous researchers have noted that heavy workloads and rapid patient turnovers can affect time allocation and care prioritization, often emphasizing acute and technical tasks over patient education (Boyde, Tuckett, and Ty 2021; Pueyo‐Garrigues et al. 2022; See et al. 2020; Wang et al. 2024). Additionally, our study provides new evidence that isolating patient education from routine activities can increase staff workload and hinder its seamless integration into clinical practice. Moreover, a novel finding from our research highlights the challenge professionals face in identifying educational needs, which were reported to be more difficult to discern than clinical needs, frequently leading to delays in initiating educational interventions.

Considering these challenges, participants in our study outlined various strategies to enhance patient education during hospitalization, identifying significant room for improvement.

Specifically, regarding staff strategies, enhancing pedagogy skills among healthcare professionals is critical to improve patient education, as supported by previous research (Fawkes and Moore 2019; Spann et al. 2024).

A recent study showed that the lack of training was one of the main hindering factors for patient education (Pueyo‐Garrigues et al. 2022). Nurses interviewed in a previous study reported the need to know the key concepts and contents and how to recognize learning styles to deliver personalized patient education (Boyde, Tuckett, and Ty 2021; Fawkes and Moore 2019; Spann et al. 2024). Indeed, professionals should be up‐to‐date on scientific information and exhibit appropriate attitudes and relational and pedagogical skills, structuring and assessing patient education (Ghorbani et al. 2019; Loyal et al. 2023).

Our study provides new insights from nurse managers on the importance of managerial strategies for enhancing patient education. These include increasing staff numbers, reducing workloads, ensuring a balanced mix of novice and experienced personnel in each shift, and introducing expert roles such as case managers to support the discharge process. In addition, managers value the potential contribution of engaging expert patients to promote peer‐education initiatives.

Nurse managers also highlighted the importance of optimizing the educational environment by designating specific spaces within wards for patient education, as supported by previous studies (Boyde, Tuckett, and Ty 2021; Ghorbani et al. 2019). Factors such as constant noise and lack of privacy were reported to distract patients and inhibit their ability to express themselves, thus hindering effective learning (Boyde, Tuckett, and Ty 2021; Fawkes and Moore 2019).

Furthermore, nurse managers in our study drew new attention to the early identification of vulnerable patients, families, and informal caregivers to improve patient education. Increasing their involvement in clinical pathways and ward activities could improve educational outcomes and help address cultural or linguistic barriers. These could be further improved by ameliorating the tools supporting patient education, especially risk assessment tools and the development of videos and digital strategies.

Lastly, many managers stressed the importance of structured yet personalized documentation of patient education. While some acknowledged that documentation can be burdensome for staff, a lack of structured documentation may lead professionals to undervalue patient education, as noted into previous studies (See et al. 2020; Spann et al. 2024). Inadequate documentation can also undermine a multiprofessional approach to achieving shared goals in caring for patients.

Linked to the latter point, our participants highlighted the significance of collaborative practice in improving the quality of patient education. According to previous research (Wang et al. 2024), our findings highlight the role of interdisciplinary collaboration in facilitating the exchange of ideas and harmonizing educational practices across professionals. However, our study brings new attention to the contribution of newly hired staff and the importance of valuing input from all healthcare team members. New insights emerged also on the importance of collaborative multidisciplinary networks between hospitals and primary care as essential for improving patient education and a safe discharge. Ensuring seamless transitional care and continuity across settings is vital for addressing clinical, educational, and social needs, especially in older people.

Integrated, multidisciplinary interventions that prioritize coordination and patient education have the potential to reduce readmission rates among geriatric patients without increasing costs (Morkisch et al. 2020).

This study has both limitations and strengths. Although it was conducted solely in medical and surgical wards, potentially limiting the generalizability of its findings to other healthcare settings, these areas account for a substantial portion of hospitalized patients and serve as key sites for patient education. By focusing on medical and surgical wards, the study provides a rich, context‐specific understanding of patient education practices in settings where a wide range of acute and chronic conditions are managed, making the findings particularly relevant to high‐demand environments. Additionally, only nurse managers were interviewed, excluding perspectives from physician managers. Nevertheless, 28 nurse managers from 34 wards were interviewed offering an in‐depth and expansive exploration of their perspective on patient education. Their insights capture the nuanced ways in which nurse managers navigate the contextual and organizational factors that influence patient education. This focused approach provides a depth of understanding that might not have been achievable with a broader but less targeted sample.

## 5 Conclusion

In summary, this study explores patient education in hospitalized adults through the lens of nurse managers. We found that nurse managers recognize patient education as a multidisciplinary process benefiting patients, families, and informal caregivers by potentially reducing patient's stress, lowering rehospitalization rates, and enhancing postdischarge self‐care. However, the emphasis often remains on information transfer and technical skills, with less focus on psychosocial aspects. Barriers such as time constraints, workload pressures, and unstable clinical conditions hinder effective patient education, often delaying it until discharge and contributing to increased readmissions.

Future research should investigate how contextual factors related to patient characteristics and hospital ward environments influence the delivery of patient education. Specifically, studies could focus on identifying how characteristics such as patient acuity, type of medical condition, patient flow metrics, and particular needs within medical or surgical wards affect the timing, methods, and effectiveness of educational interventions.

## 6 Relevance for Clinical Practice

To improve and expand patient education, organizational support and clinical support are crucial. This includes support from nurse managers in prioritizing patient education alongside clinical tasks, investing in staff training, optimizing the skill mix, developing digital educational tools, and structured documentation. Training programs for professionals should encompass not just clinical aspects, but also pedagogical skills and psychosocial elements to boost patient involvement early in hospitalization, particularly in medical wards. Adding professionals with expertise in education in the team or across wards and incorporating input from frontline staff can further enhance educational interventions. Additionally, nurse managers at all levels should promote the optimization of educational tools and documentation and develop multimedia resources like videos that can improve the effectiveness of patient education. Structured approaches can help professionals deliver content more effectively and enable patients to better appreciate its value. However, a mixed approach combining structured protocols and informal education could be considered to ensure a high quality of patient education while respecting patient's needs and conditions. Establishing strong multidisciplinary networks, especially between hospitals and primary care, as part of structure policy and nurse managers' relationships can further support patient education efforts. All these efforts can improve patients' self‐care and informal caregivers' abilities, reducing rehospitalization rates, length of hospitalizations, and related costs.

## Author Contributions

**Jessica Longhini:** conceptualization, writing – original draft, data curation, formal analysis. **Elisa Ambrosi:** writing – review and editing, methodology, supervision. **Barbara Tescaro:** supervision, formal analysis, conceptualization, writing – review and editing. **Noemi Derugna:** conceptualization, data curation, formal analysis, writing – original draft. **Maria Luisa Ferro:** writing – original draft, conceptualization, formal analysis, data curation. **Federica Canzan:** supervision, writing – review and editing, methodology.

## Ethics Statement

The study has been approved by the Comitato di Approvazione della Ricerca sulla Persona (N. 28_2023).

## Consent

Informed signed consent has been collected from participants. Data have been managed anonymously, ensuring privacy criteria according to GDPR.

## Conflicts of Interest

The authors declare no conflicts of interest.

## Data Availability

The data that support the findings of this study are available from the corresponding author upon reasonable request.

## Appendix A

### Interview Guide

Information

- Age
- Gender
- Years of experience as a nurse
- Years of experience as a nurse manager
- Type of ward managed
- Number of health professionals coordinated

### Interview Questions

When it comes to therapeutic education, what comes to mind? How would you describe it?

- In‐depth questions if it does not emerge from the narration:
  ○ In your opinion, how important is therapeutic education?
  ○ In your opinion, why is it necessary to do therapeutic education?
  ○ Could you provide an example of patient education you saw performed by health professionals?

How is patient education carried out in your department?

- In‐depth questions if it does not emerge from the narration:
  ○ Who carries it out?
  ○ At what times is it carried out? (time of the shift, stage of hospitalization)
  ○ What does the educational moment consist of? Could you provide examples?
  ○ How long does an education session normally take?
  ○ Who are the most frequent recipients?
  ○ With respect to caregivers and family members, how is education carried out?
  ○ Is education discussed in the handovers?
  ○ What do you think is the level of quality of education that nurses provide?

Do you think there are any factors that facilitate or hinder educating a patient?

- In‐depth questions if it does not emerge from the narration:
  ○ What are the facilitating elements? Difficulties?
  ○ What are the difficulties you encounter in facilitating the development of education?
  ○ What are the strategies that you, as manager, have put into practice to promote patient education?
  ○ Do you think there are areas for improvement with respect to education?
  ○ What could facilitate patient education?
